# Relationship between the Second to Fourth Finger Length Ratio and Calcaneus Quantitative Ultrasound

**DOI:** 10.1038/s41598-018-33056-z

**Published:** 2018-10-02

**Authors:** Yoko Takahata, Kumi Hirokawa

**Affiliations:** Faculty of Nursing, Baika Women’s University, Ibaraki, Osaka 567-8578 Japan

## Abstract

This study aimed to characterize the relationship between the ratio of the length of the second and fourth fingers (2D:4D value) and the speed of sound of the calcaneus by quantitative ultrasound (QUS-SOS) in undergraduate female students. We recruited 138 young women with a mean age of 19.6 ± 1.4 years. The participants’ calcaneus QUS-SOS was measured using an ultrasound bone densitometer. We also measured the participants’ weight, height, and grip strength. A self-reported questionnaire was used to obtain information on participants’ secondary sexual characteristics, and exercise habits. The present study showed that the 2D:4D value of both hands was significantly correlated with the calcaneus QUS-SOS. The 2D:4D value of the left hand was also positively associated with the calcaneus QUS-SOS results in several respects. These findings suggest that the 2D:4D value may be useful for the screening of risk for a low bone quality in undergraduate female students.

## Introduction

A proper and balanced modeling and remodeling cycle of bone is the key to reducing breakability, attaining sufficient peak bone density and reducing the risk of osteoporosis in later life. As the bone turnover system slows with age, it is important to ensure sufficient bone development in childhood and adolescence^1–3^.

How the biochemical markers of bone turnover affect the remodeling of the bone and changes in different age ranges and in obesity has been examined physiologically. Walsh *et al*. reported that the levels of biochemical markers peak in the middle of puberty and then decrease in late puberty^4^. There has been some discussion about the relationship between exercise and bone density, including such factors as bone mineral density, bone mineral content, and the stiffness index of the calcaneus by quantitative ultrasound (QUS-SI)^5–9^. Regular menstruation is also important for females, as the gonadal hormones, including estrogen, are known to affect the remodeling of bones and reduce their breakdown; indeed, estrogen is administered as a treatment for osteoporosis^10,11^. Ensuring a certain level of muscle mass and moderate fat mass is necessary to earn higher quantitative ultrasound (QUS) values^12^.

Sex-based differences in the ratio of the length of the second and fourth fingers (2D:4D value) have been investigated, and males have shown to have a lower 2D:4D value than females^13^. This is because the 2D:4D value is influenced by the level of sex hormones such as testosterone and estrogen in fetal period^13^. Moreover, Knickmeyer *et al*. reported that the levels of sex hormones have a continuous influence until the early postnatal period^14^. Manning *et al*.^13^ reviewed the evidence that the sexual dimorphism in 2D:4D develops in utero in humans and Zheng and Cohn^15^ have provided experimental evidence that this is so in mice. The sex difference arises because digit 4 has high numbers of androgen and estrogen receptors and male fetuses in general tend to produce more testosterone than female fetuses. Furthermore, it has been shown that 2D:4D in pre-menopausal women is correlated with estrogen levels across the cycle^16–19^. Knickmeyer *et al*. pointed out that these sex differences exist in children as young as two years of age^14^. The maternal 2D:4D value also found to correlate positively with the 2D:4D value in daughters^20^, and high prenatal levels of androgen lead to fetuses with a low 2D:4D value^14^. According to these previous studies, the sexually dimorphic 2D:4D value begins to manifest during embryonic development and depends on the levels of androgen or estrogen in the fetus and in the mother.

The levels of bone turnover markers (which are related to bone density) of daughters are likely influenced by their mothers^21^. Su *et al*. also reported that the development of bone is affected by the gonadal hormone levels in mothers during pregnancy^22^. Both the 2D:4D value and bone density are affected during the embryonic or fetal period by the hormone levels of pregnant women, thus suggesting that these values may be correlated. Hypothesizing that the bone density was decided prenatally, at least in part, we attempted to characterize the relationship between the 2D:4D value and the speed of sound of the calcaneus by quantitative ultrasound (QUS-SOS) to determine whether or not the 2D:4D value may be a useful factor for screening undergraduate female students who are at risk of having a poor bone quality.

## Results

### Participants’ characteristics

The participants’ characteristics such as age, 2D:4D on both hands, the calcaneus QUS status, height, weight, body fat percentage, body mass index (BMI), grip strength, and the age at menarche, are listed in Table 1. A total of 63% of participants reported exercise habits when they were junior high school students, but this value dropped to about 30% in high school.

**Table 1:** Subject characteristics, n = 138.

	Mean ± SD (range)
Age (years)	19.6 ± 1.4 (18–22)
2D:4D on the right hand	0.9716 ± 0.03 (0.85–1.08)
2D:4D on the left hand	0.9545 ± 0.03 (0.88–1.06)
Calcaneus QUS-SI (Stiffness index)	103.3 ± 15.2 (70.0–156.0)
Calcaneus QUS-SOS (m/s)	215.2 ± 52.4 (91.0–255.0)
Calcaneus QUS-BUA (dB/MHz)	32.5 ± 1.5 (31.0–41.0)
Height (cm)	157.1 ± 5.4 (142.9–172.3)
Weight (kg)	50.5 ± 6.9 (37.5–73.5)
Body Fat Percentage (%)	26.1 ± 4.4 (12.2–39.3)
BMI (kg/m^2^)	20.4 ± 2.3 (15.0–29.0)
Grip strength (kg)	29.8 ± 5.7 (15.0–45.0)
Secondary sexual characteristic development* (age in years)	12.2 ± 1.5 (9.0–16.1)
	n (%)
Exercise experience in junior high school	88 (63.8%)
Exercise experience in high school	50 (36.2%)

2D:4D, ratio of the finger length of the 2^nd^ and 4^th^ digit; BMI, body mass index; QUS-SI, stiffness index by quantitative ultrasound;

QUS-SOS, speed of sound by quantitative ultrasound; QUS-BUA, broadband ultrasound attenuation by quantitative ultrasound.

^*^The age at which menstruation started.

### Relationship of the 2D:4D value of both hands on the calcaneus QUS values in young females

The relationship between the 2D:4D value and the calcaneus QUS values was analyzed using Pearson’s correlation coefficient. The results of these analyses are shown in Table 2. There was a significant correlation between the 2D:4D value of both hands and the calcaneus QUS-SOS.

**Table 2:** The effect of the digit ratio (2D:4D) of both hands on the calcaneus QUS values with Pearson’s correlation coefficient, n = 138.

	2D:4D of the right hand	2D:4D of the left hand
2D:4D of the right hand	—	*r* = 0.649
		*p* = 0.000*
2D:4D of the left hand	*r* = 0.649	—
	*p* = 0.000*	
Calcaneus QUS-SOS	*r* = 0.155	*r* = 0.198
	*p* = 0.035*	*p* = 0.010*
Calcaneus QUS-BUA	*r* = 0.109	*r* = 0.037
	*p* = 0.102	*p* = 0.333
Calcaneus QUS-SI	*r* = 0.084	*r* = 0.061
	*p* = 0.164	*p* = 0.238
Grip strength	*r* = 0.018	*r* = 0.044
	*p* = 0.830	*p* = 0.608
BMI	*r* = 0.008	*r* = −0.037
	*p* = 0.928	*p* = 0.663
Secondary sexual	*r* = 0.034	*r* = 0.067
characteristic development^†^	*p* = 0.693	*p* = 0.438

2D:4D, ratio of the finger length of the 2^nd^ and 4^th^ digit; QUS-SI, stiffness index by quantitative ultrasound;

QUS-SOS, speed of sound by quantitative ultrasound; QUS-BUA, broadband ultrasound attenuation by

quantitative ultrasound; BMI, body mass index.

^†^The age at which menstruation started.

^*^Indicates a significant correlation with calcaneus QUS-SOS.

### Relationship of the 2D:4D value of both hands, BMI, and exercise habit on the calcaneus QUS values in young females

The relationship of the 2D:4D value, BMI, and exercise habit with the calcaneus QUS values was analyzed using a multiple regression analysis after adjustment for age. The results of these analyses are displayed in Table 3. The findings are explained in detail alongside the results for each calcaneus QUS value below.

**Table 3:** The effect of the digit ratio (2D:4D) of both hands on the calcaneus QUS values by a multiple regression analysis, n = 138.

	Factors	β	*p* values
Calcaneus QUS-SOS	2D:4D on the right hand	0.167	0.052
	Grip strength (kg)	0.003	0.974
	Exercise experience in junior high school	0.008	0.937
	Exercise experience in senior high school	0.155	0.122
	Secondary sexual characteristic development* (age in years)	−0.017	0.850
	BMI (kg/m^2^)	0.070	0.455
	***F***	1.253	0.284
	***R-squared/Adjusted R-squared***	0.054/0.011	
	2D:4D on the left hand	0.215	*0.013* ^†^
	Grip strength (kg)	0.006	0.946
	Exercise experience in junior high school	0.007	0.943
	Exercise experience in senior high school	0.165	0.096
	Secondary sexual characteristic development* (age in years)	−0.020	0.821
	BMI (kg/m^2^)	0.081	0.385
	***F***	1.688	0.129
	***R-squared/Adjusted R-squared***	0.072/0.029	
Calcaneus QUS-BUA	2D:4D on the right hand	0.116	0.178
	Grip strength (kg)	0.115	0.206
	Exercise experience in junior high school	0.076	0.450
	Exercise experience in senior high school	0.037	0.714
	Secondary sexual characteristic development* (age in years)	−0.140	0.127
	BMI (kg/m^2^)	0.052	0.579
	***F***	1.096	0.368
	***R-squared/Adjusted R-squared***	0.004/1.507	
	2D:4D on the left hand	0.038	0.659
	Grip strength (kg)	0.116	0.207
	Exercise experience in junior high school	0.075	0.457
	Exercise experience in senior high school	0.030	0.766
	Secondary sexual characteristic development* (age in years)	−0.138	0.137
	BMI (kg/m^2^)	0.049	0.602
	***F***	0.813	0.561
	***R-squared/Adjusted R-squared***	0.036/0.008	
Calcaneus QUS-SI	2D:4D on the right hand	0.090	0.290
	Grip strength (kg)	0.108	0.231
	Exercise experience in junior high school	0.138	0.169
	Exercise experience in senior high school	0.049	0.622
	Secondary sexual characteristic development* (age in years)	−0.083	0.363
	BMI (kg/m^2^)	0.040	0.666
	***F***	1.373	0.230
	***R-squared/Adjusted R-squared***	0.059/0.016	
	2D:4D on the left hand	0.061	0.476
	Grip strength (kg)	0.107	0.239
	Exercise experience in junior high school	0.134	0.182
	Exercise experience in senior high school	0.048	0.632
	Secondary sexual characteristic development* (age in years)	−0.082	0.368
	BMI (kg/m^2^)	0.044	0.640
	***F***	1.264	0.278
	***R-squared/Adjusted R-squared***	0.055/0.011	

2D:4D, ratio of the finger length of the 2^nd^ and 4^th^ digit; BMI, body mass index; QUS-SI, stiffness index by quantitative ultrasound;

QUS-SOS, speed of sound by quantitative ultrasound; QUS-BUA, broadband ultrasound attenuation by quantitative ultrasound.

^*^The age at which menstruation started, ^†^Indicates a significant correlation with calcaneus QUS-SOS. Adjusted for age.

Regarding the relationship of the 2D:4D value of the right hand on the calcaneus QUS-SOS by a multiple regression analysis, there was no significant association between any factors and the calcaneus QUS-SOS.

Regarding the relationship of the 2D:4D value of the left hand on the calcaneus QUS-SOS by a multiple regression analysis, a significant association was only observed between the 2D:4D value of the left hand and the calcaneus QUS-SOS (β = 0.215, p = 0.013).

Regarding the relationship of the 2D:4D value of the right hand on the broadband ultrasound attenuation of the calcaneus by quantitative ultrasound (QUS-BUA) results by a multiple regression analysis, there was no significant association between any factors and the calcaneus QUS-BUA.

Regarding the relationship of the 2D:4D value of the left hand on the calcaneus QUS-BUA results by a multiple regression analysis, there was no significant association between any factors and the calcaneus QUS-BUA.

Regarding the relationship of the 2D:4D value of the right hand on the stiffness index of the calcaneus by quantitative ultrasound (QUS-SI) results by a multiple regression analysis, there was no significant association between any factors and the calcaneus QUS-SI.

Regarding the relationship of the 2D:4D value of the left hand on the calcaneus QUS-SI results by a multiple regression analysis, there was no significant association between any factors and the calcaneus QUS-SI.

## Discussion

The present study showed that the 2D:4D values of both hands were significantly correlated with the calcaneus QUS-SOS (Table 2). The 2D:4D value of the left hand was also positively associated with the calcaneus QUS-SOS results according to a multiple regression analysis (Table 3).

The existence of sexual dimorphism in 2D:4D in humans and mice has been described by Manning *et al*.^13^ and Zheng and Cohn^15^, respectively. The sex difference in 2D:4D arises because the fetal 4^th^ digit has many androgen and estrogen receptors and males tend to produce more androgen than females. Knickmeyer *et al*. reported that the 2D:4D value generally increases the differences in both sexes with age^14^. However, this value also depends on androgen or estrogen levels in mothers during pregnancy^14^. In premenopausal women, the 2D:4D value has been reported to correlate with the estrogen level^16–19^. In addition, Richards *et al*. pointed out that the correlation should differ by menstrual cycle phase^17^. The estrogen level in women affects the bone density as well as the risk of osteoporosis and fracture^10,11^. Su *et al*. also reported that the growth of bone is affected by the gonadal hormone levels in mothers during pregnancy^22^. Based on the findings from the present study, the 2D:4D value seems to be positively associated with the QUS-SOS. This phenomenon suggests that it may be possible to screen university students with a relatively low 2D:4D value of the left hand to identify those who are at risk for a low bone density.

Takahata *et al*. found that excessive restriction of maternal weight gain might have a negative relationship on the calcaneus QUS-SI for the offspring in their study on bone health^8^. These findings suggest that maternal weight management should be considered when performing antenatal intervention. The maternal weight may also affect the 2D:4D value development during the embryonic or fetal period. The management of maternal weight may be the one of the key to clarifying the relationship between the 2D:4D value and bone density during pregnancy.

There are several limitations associated with this study. First, we used an ultrasound bone densitometer, a low-invasive technique, to measure the calcaneus QUS-SOS instead of a dual-energy X-ray absorptiometry (DXA) scan (the preferred technique for measuring the bone mineral density (BMD)) because the participants were relatively young. Second, we were unable to prove a cause-and-effect relationship between the 2D:4D value and the calcaneus QUS-SOS results because of the cross-sectional nature of our study. Third, other patient-related factors such as the exercise experience, might have affected the results of the present study. Further research may help confirm the 2D:4D value to be an influential factor for the QUS-SOS in undergraduate female students.

## Conclusion

We characterized the relationship between the 2D:4D value and the calcaneus QUS-SOS in undergraduate female students. These findings suggest that the 2D:4D value may be a useful indicator for screening the calcaneus QUS-SOS value. Furthermore, our findings also suggest that a high estrogen level affects the bone density positively, according to Table 3. Prenatal testosterone levels can therefore affect the bone development in later life. Further longitudinal studies are needed to confirm the present findings.

## Materials and Methods

### Participants

We estimated the sample size using G Power 3.1. Based on the result, 107 as was considered to be an appropriate sample size for the multiple regression analysis. We recruited 141 undergraduate female students and recorded their anthropometric data from November 2013 to October 2015. Two participants (1.4%) were excluded from the study because we could not measure their calcaneus bone values due to them having allergies to the ethanol for disinfection used during measurements; one subject (0.7%) was also excluded because her age did not fit this study. 138 participants (97.9%) were in good health and were free of chronic diseases affecting the bone metabolism. Written informed consent was obtained from all participants in accordance with the Declaration of Helsinki. The study was approved by the Ethics Committee of Baika Women’s University and all experiments were performed in accordance with relevant guidelines and regulations.

### Calcaneus bone density measurements

The calcaneus QUS-SOS, the calcaneus QUS-BUA, and the calcaneus QUS-SI were measured using an ultrasound bone densitometer (Achilles InSight; GE Healthcare, Little Chalfont, UK). The calcaneus QUS-SOS was used to assess bone density in this study since this value is more important for supporting the value of the calcaneus QUS-SI than the calcaneus BUA and is also used as an indicator for bone density and clinically as an evaluation index. We measured the calcaneus which mostly consists of cancellous bone. If such cancellous bone is frangible, then it will often cause osteoporosis. We used an ultrasound bone densitometer because it has no side effects and correlates well with the DXA-measured BMD^23,24^ or bone mineral contents (BMC)^23^. The densitometer was operated by one researcher experienced in taking such measurements.

### Physical examinations

The participants’ height (determined with the DST-210N; Muratec-KDS Corp., Kyoto, Japan) and weight, body fat percentage (determined with the DC-320; Tanita Corp., Tokyo, Japan) were measured after shoes were removed, and then we calculated BMI. A Smedley type dynamometer was used to measure grip strength, which represented the muscle strength^25,26^. All measurements were performed twice for each hand, and the highest result for each hand was used to calculate the mean grip strength.

### The 2D:4D value

The 2D:4D value was measured after scanning the image of both hands of each subject using a scanner (CanoScan LiDE 200; Canon, Tokyo, Japan). The digit length was measured on the ventral surface of the hand from the basal crease of the digit to the tip, using the Vernier Scale based on the findings of the scanning image on paper. We then calculated the ratio of the length of the second to fourth digits on both hands.

### Questionnaire

Specific details asked about in the self-reported questionnaire were as follows: Date of birth, age at which menstruation started, exercise habits in junior high school (including the sports name), and exercise habits in high school (including the sports name). The age of onset of secondary sexual characteristics was determined based on the age at which menstruation started. The questions were explained to the participants if any item on their questionnaire was left unanswered.

### Statistical analyses

Pearson’s correlation coefficient was used to determine the relationship between the 2D:4D value and the calcaneus QUS values, and a multiple regression analysis was used to determine the relationships of the 2D:4D value on the calcaneus QUS values. The level of suitability to the model was shown as R-squared in each model. A p-value of <0.05 was considered to be statistically significant. Statistical analyses were performed using the IBM SPSS 23.0 software program (IBM Corp. Released 2015. IBM SPSS statistics for Windows, Version 23.0. Armonk, NY, USA.).
